# Viral Infections of the Vulva: A Narrative Review

**DOI:** 10.3390/life15091365

**Published:** 2025-08-28

**Authors:** Matteo Terrinoni, Tullio Golia D’Augè, Ottavia D’Oria, Michele Palisciano, Federica Adinolfi, Dario Rossetti, Gian Carlo Di Renzo, Andrea Giannini

**Affiliations:** 1 Department of Medicine and Surgery, University of Perugia, 06129 Perugia, Italy; 2 Department of Obstetrics and Gynecology, “Alto Tevere” Hospital of Città di Castello, USL Umbria 1, 06127 Perugia, Italy; dario.rossetti@uslumbria1.it; 3 Ospedale San Pietro Fatebenefratelli, 00189 Rome, Italy; tullio.goliadauge@uniroma1.it; 4 Obstetrics and Gynecological Unit, Department of Woman’s and Child’s Health, San Camillo-Forlanini Hospital, 00152 Rome, Italy; ottavia.doria@uniroma1.it; 5 Department of Obstetrics and Gynecology, “Branca” Hospital of Gubbio-Gualdo Tadino, USL Umbria 1, 06127 Perugia, Italy; michele.palisciano@live.it; 6 Department of Obstetrics and Gynecology, Azienda Ospedaliera di Perugia, 06129 Perugia, Italy; federica.adinolfi@ospedale.perugia.it; 7 PREIS School (International and European School of Perinatal, Neonatal and Reproductive Medicine), 50121 Florence, Italy; profgcdr@gmail.com; 8 Department of Obstetrics, Gynecology and Perinatology, I.M. Sechenov First State University of Moscow, 119991 Moscow, Russia; 9 Unit of Gynecology, Sant’Andrea Hospital, Department of Surgical and Medical Sciences and Translational Medicine, Sapienza University of Rome, 00189 Rome, Italy; andrea.giannini@uniroma1.it

**Keywords:** genital herpes, HPV, molluscum contagiosum, vaccination, viral infections, vulva

## Abstract

Vulvar viral infections such as condyloma acuminata, genital herpes, molluscum contagiosum, and Lipschütz ulcers span both sexually and non-sexually transmitted diseases and affect patients across all age groups. Lesions may present as papules, verrucous growths, or painful ulcers, often causing functional impairment and significant psychosocial distress. A multidisciplinary strategy that integrates epidemiology, precise diagnostics, individualized therapy, and psychological support is essential to optimize outcomes. We performed a structured literature search in PubMed, Scopus, and Web of Science using terms “vulvar viral infection,” “HPV,” “HSV,” “molluscum contagiosum,” and “Lipschütz ulcers.” International guidelines from the UK, Europe, and Australia were reviewed, alongside reference lists of key articles. Particular attention was given to paradoxical presentations, pediatric considerations, and cost-effectiveness analyses. HPV vaccination programs have markedly reduced anogenital warts, while early PCR/NAAT for HSV accelerates targeted antiviral therapy. First-line treatments like oral acyclovir/famciclovir for HSV and topical imiquimod or podophyllotoxin (±cryotherapy) for HPV are supported by adjunctive measures for self-limiting conditions. Host factors (hormonal cycles, immune status) and local irritants modulate recurrence risk, informing anticipatory suppressive regimens and barrier-reinforcing care. Validated patient-reported outcome measures (VPAQ, DLQI, FSFI) capture pain, sexual function, and quality-of-life impacts. Health–economic evaluations underscore the long-term value of rapid diagnostics and broad vaccination. Personalized, multidisciplinary management that combines prevention, precision diagnostics, tailored therapy, psychosocial support, and economic considerations offers the greatest promise for improving clinical and quality-of-life outcomes in patients with vulvar viral infections. We aim to outline best practices for the diagnosis and management of common vulvar viral infections, providing practical guidance for clinicians to improve recognition and therapeutic decision-making.

## 1. Introduction

Vulvar viral infections are a large group of pathologies, including both sexually transmitted and non-sexually transmitted diseases. They can affect both younger and older patients and could manifest in many ways, from well-demarcated papules to painful ulcers with functional impairment. Condyloma acuminata, genital herpes, molluscum contagiosum, and Lipschütz ulcers are the most common clinical conditions and require clinicians to integrate epidemiological data, diagnostic modalities, and individualized therapy [1,2,3,4].

This review aims to synthesize current evidence on epidemiology, diagnosis, and management of key vulvar viral infections with the aim of providing an overall vision that supports clinicians in their diagnostic and decision-making process, highlighting areas for future research.

## 2. Materials and Methods

We conducted an enlarged structured search of PubMed, Scopus, and Web of Science using terms “vulvar viral infection,” “HPV,” “HSV,” “molluscum contagiosum,” and “Lipschütz ulcers.” International guidelines (UK, European, Australian) and reference lists of relevant articles were also reviewed. We integrated particular and paradoxical cases that could be encountered in clinical practice. We also reserved a specific section for the pediatric population.

## 3. Results

### 3.1. Epidemiology and Pathogenetic Aspects

#### 3.1.1. Condyloma Acuminata (HPV)

Human papillomaviruses infect the basal layer of stratified squamous epithelium after access is gained through micro-abrasions. Following entry, HPV establishes an infection tightly linked to epithelial differentiation: early viral genes (E1, E2, and, for high-risk types, E6/E7) drive replication and modulate host cell cycle control to allow productive replication in suprabasal keratinocytes while delaying cell death. Low-risk genotypes (principally 6 and 11) typically cause benign wart formation by promoting hyperproliferation without marked genomic instability; high-risk genotypes (e.g., 16, 18, 31) express oncoproteins (E6/E7) that interfere with p53 and Rb pathways and therefore carry risk of dysplastic progression when present in transformation-prone mucosa. HPV evades innate immunity by limiting cytopathic effects and by interfering with interferon signaling and antigen presentation, which facilitates persistence. In Western countries, condyloma acuminata is among the most common genital viral infections. Italian surveillance showed rising incidence from 2000 to 2016, followed by a decline linked to HPV vaccination campaigns [5,6,7]. Local host factors, including mucosal barrier integrity, microbiome composition, hormonal milieu, and systemic immune competence, modulate both acquisition and persistence of infection. In immunocompromised patients (e.g., HIV, transplant recipients), higher viral loads, multicentric disease, rapid recurrence, and an increased likelihood of high-risk genotype co-infections are commonly observed, justifying more intensive genotyping (including high-risk types 16, 18, 31, and 33) and surveillance in these populations [8,9].

#### 3.1.2. Genital Herpes (HSV)

HSV infects mucoepithelial cells where it undergoes lytic replication, producing clustered vesicles and erosions. Viral entry is mediated by viral glycoproteins interacting with cellular receptors (e.g., nectin family, HVEM), followed by replication in the epithelium and retrograde transport of virions to sensory ganglia, where latency is established. During latency, viral genomes persist in neuronal nuclei with restricted transcription (latency-associated transcripts). Reactivation (and anterograde axonal transport to the mucosa) is triggered by diverse stimuli like stress, febrile illness, hormonal fluctuations, local trauma, and UV exposure that produce recurrent mucocutaneous lesions. They are caused by HSV-2, but HSV-1 is increasingly implicated. It predominantly affects young, sexually active women. Recent data suggest modest declines at some centers, possibly reflecting better prevention and management [10,11,12]. The host immune response is a critical determinant of clinical severity and duration: innate sensors (TLRs, intracellular nucleic acid receptors), NK cells, and robust CD8^+^ T-cell responses limit spread, while local inflammatory mediators produce pain and ulceration. HSV has evolved immune-modulatory strategies (e.g., inhibition of antigen presentation and interferon pathways) that allow both frequent subclinical shedding and symptomatic recurrence clinical severity [13,14].

#### 3.1.3. Molluscum Contagiosum

Molluscum contagiosum virus (a poxvirus) replicates exclusively in the cytoplasm of epidermal keratinocytes. Infection produces characteristic molluscum (Henderson–Patterson) bodies characterized by large intracytoplasmic inclusion bodies composed of virions and viral proteins that eventually form the central umbilicated core (“dimple”). The virus tends to induce a limited inflammatory response early on, which permits persistence and autoinoculation; in immunocompromised hosts, lesions can be numerous, confluent, and atypical. The viral life cycle and modulation of innate immune responses underlie the protracted course seen in some patients [11,15].

#### 3.1.4. Lipschütz Ulcers

Lipschütz (acute vulvar) ulcers are generally considered a non-sexually transmitted, acute, and typically self-limited syndrome most often occurring in adolescents and young women. Current evidence supports a post-infectious immunologic mechanism in many cases: a systemic viral (or, less commonly, bacterial) trigger precedes the ulcerative episode and a robust local inflammatory response ensues, producing painful necrotic ulcers (“kissing ulcers”). Proposed mechanisms include immune complex formation, neutrophil-dominant inflammation, and localized microvascular injury; associations with EBV, CMV, and, recently, SARS-CoV-2 have been reported as potential precipitants. Importantly, because the pathogenesis is frequently immune-mediated rather than direct cytolysis by a genital pathogen, management focuses on symptomatic control and exclusion of specific transmissible causes, which resolve in 2–3 weeks without scarring [4,16,17,18].

#### 3.1.5. Host Factors and Modifying Influences

Across these viral entities, key host-level determinants (hormonal cyclical changes, mucocutaneous microenvironment, the local microbiome, and systemic immune status) interact with viral properties (tropism, latency strategies, and immune evasion) to determine clinical phenotype, recurrence risk, and response to therapy. This interaction explains, for example, catamenial HSV recurrences, more severe HPV disease in immunosuppressed patients, and atypical molluscum in those with impaired cellular immunity [19,20].

### 3.2. Clinical Presentation and Diagnosis

#### 3.2.1. Condyloma Acuminata (HPV)

- Morphology: papular, verrucous, or sessile lesions; may be flat or exophytic, and when confluent can form larger “cauliflower-like” plaques. Surface may bleed with friction. Sizes range from millimetric papules to centimetric masses.
- Symptoms: often asymptomatic or mildly pruritic; secondary irritation and malodor may occur with large lesions. In pregnancy, lesions can enlarge due to physiologic immune modulation.
- Typical course: variable persistence; spontaneous regression is possible, particularly in the immunocompetent [21].

#### 3.2.2. Genital Herpes (HSV)

- Morphology and evolution: small, grouped vesicles on an erythematous base that rapidly ulcerate. Primary infections are often more extensive and accompanied by systemic symptoms (fever, malaise, myalgias), while recurrences are usually smaller and localized. Associated regional tender lymphadenopathy is common.
- Prodrome: tingling, burning, or paresthesia is frequently reported hours to days before lesion appearance.
- Timeline: primary lesions often crust and heal over 2–3 weeks without scarring; recurrences are shorter (typical healing 5–10 days), especially if antiviral therapy is started early.
- Pain: pain and dysuria are common; sexual activity is often painful during active lesions [10].

#### 3.2.3. Molluscum Contagiosum

- Morphology: small (2–5 mm), dome-shaped, flesh-colored papules with central umbilication; a curd-like plug is sometimes expressed from the central pore. Lesions may be solitary or multiple and frequently auto-inoculate along lines of trauma.
- Symptoms: typically asymptomatic or mildly pruritic; in children, often multiple but not painful. In adults, genital molluscum is frequently sexually transmitted [11].

#### 3.2.4. Lipschütz Ulcers

- Morphology: one or more deep, often symmetric ulcers commonly on the labia minora or vestibule; bases can be necrotic or fibrinous with sharply demarcated edges (“kissing” ulcers when symmetric).
- Symptoms and systemic features: severe pain is typical, often accompanied by fever, malaise, and cervical or inguinal adenopathy. Healing is usually spontaneous over 2–3 weeks but may require analgesia and wound care [4,16,17,18].

#### 3.2.5. Dermatoscopy and Bedside Clues

Dermatoscopy can help differentiate warts (papilliform surface, thrombosed capillaries) from other papular lesions; molluscum shows a central pore and characteristic polylobular structures; grouped vesicles or erosions with clustered distribution suggest herpes. These noninvasive clues can accelerate diagnostic reasoning at the bedside [11,21,22,23].

#### 3.2.6. Diagnostic Modalities

- HSV testing: PCR/NAAT on lesion swab (prefer sampling fluid from unroofed vesicles or swabbing the base of an ulcer) is the diagnostic gold standard for acute lesions because of high sensitivity and rapid turnaround. Serology may have a role in retrospective determination of type-specific exposure, but is less useful for acute lesion management. Timing matters: PCR sensitivity is highest early in the lesion lifecycle [21,22].
- HPV testing: routine HPV DNA testing is generally not required for typical external condyloma but is indicated in atypical, refractory, or multifocal disease and in immunocompromised patients in whom genotyping informs dysplasia surveillance. If a biopsy is performed, histology will show papillomatosis and koilocytosis in HPV-related lesions.
- Molluscum: generally clinical diagnosis; reserve biopsy or histology (showing molluscum bodies) for atypical lesions or immunocompromised hosts [11].
- Lipschütz ulcers: a diagnosis of exclusion; recommended testing includes PCR/NAAT for HSV and VZV on lesion swabs, serologies, and testing for syphilis and HIV as indicated, and consideration of EBV/CMV testing when systemic prodrome suggests these triggers. Biopsy is rarely required but may be considered when ulcers are atypical or do not heal as expected [11,21,22,23].

#### 3.2.7. Differential Diagnosis

**Figure 1:**
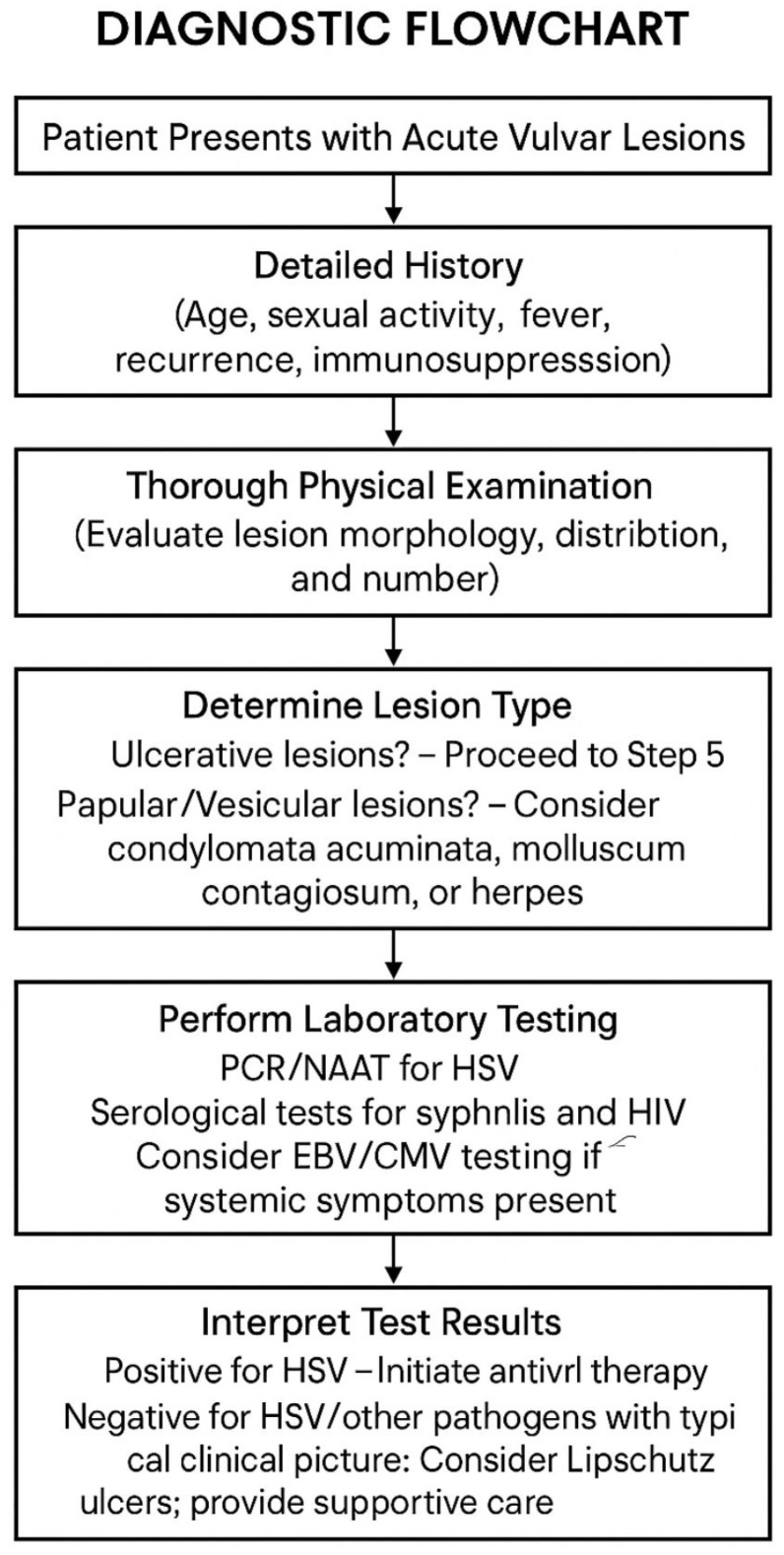
Diagnostic flowchart.

- Ulcerative lesions: distinguish painful herpetic ulcers and Lipschütz ulcers from painless syphilitic chancres (firm, indurated, typically solitary), aphthous ulcers, Crohn’s-related fissures, or fixed drug eruptions.
- Papular lesions: differentiate condyloma from molluscum, seborrheic keratoses, angiokeratomas, and early squamous intraepithelial lesions (consider biopsy if lesion is suspicious for neoplasia).
- The differential diagnosis also includes the exclusion of bacterial infections (e.g., bacterial vaginosis) and other dermatological causes such as lichen planus or lichen sclerosus, which may coexist [21,22,23,24] (Table 1) (Figure 1).

Legend—Steps: (1) Initial assessment: focused history and physical to assess urgency and risk; (2) Lesion-type determination: classify ulcerative vs. vesicular/papular/warty; (3) Sampling/testing: targeted lesion swabs (e.g., HSV PCR), serology (syphilis, HIV) and other tests as indicated; (4) Interpretation: integrate results with the clinical picture; (5) Treatment selection: start pathogen-directed therapy and symptomatic care; (6) Follow-up: monitor response, re-test or refer if worsening.

#### 3.2.8. Paradoxical Cases

In the differential diagnosis of vulvar ulcers, it is crucial to consider paradoxical reactions such as Behçet’s disease induced by IL-17A inhibitors. Although this entity does not typically fall within the spectrum of viral infections, its inclusion underscores the importance of evaluating a patient’s pharmacological history and excluding noninfectious causes in the presence of vulvar ulcers [25,26]. For instance, paradoxical Behçet’s disease—characterized by recurrent oral and genital ulcers—has been reported in patients undergoing treatment with IL-17A inhibitors like ixekizumab and secukinumab for psoriasis or other immune-mediated diseases [27,28]. Recognizing this possibility helps clinicians avoid misdiagnosis and guides them toward discontinuing the inciting drug and instituting appropriate therapy.

#### 3.2.9. Consideration of Pediatric Presentations

The Royal Children’s Hospital Melbourne guidelines recommend a structured, five-domain approach to pediatric genital ulceration (viral, bacterial/fungal, autoimmune/inflammatory, traumatic/mechanical, and paradoxical/drug-related) augmented by special vigilance in immunocompromised or atypical cases. Clinicians should take a detailed history (including systemic symptoms, medication exposures, and family autoimmune patterns) and perform a thorough examination of ulcer morphology and extra-genital findings. Targeted investigations like PCR for herpesviruses and syphilis, bacterial/fungal cultures, serologies (EBV, CMV, ANA, HLA-B51), inflammatory markers, and, when indicated, biopsy may help distinguish common non-sexually acquired infections (e.g., HSV, impetigo), recurrent aphthous or Behçet’s-related ulcers, Crohn’s fissures, and drug eruptions. In immunosuppressed children, the threshold for systemic antivirals, evaluation for opportunistic pathogens, and early specialist referral should be lower to ensure prompt diagnosis and management [29].

#### 3.2.10. Technique and Timing Recommendations

- For vesicular lesions: unroof vesicle and swab base → send for PCR/NAAT.
- For crusted or ulcerated lesions: vigorously swab ulcer base and edge; consider repeating sampling if initial tests are negative but clinical suspicion remains high.
- For biopsy: reserve for atypical lesions, suspected neoplasia, persistent refractory lesions, or when histologic confirmation will alter management.
- For immunocompromised patients: lower threshold for molecular testing, genotyping, and early specialist involvement [21,22] (Table 2).

### 3.3. Therapeutic Management and Follow-Up

Once the diagnosis is confirmed, treatment should be individualized to the type of lesion and the patient’s circumstances. For condyloma acuminata caused by HPV, first-line therapy is topical immunomodulation through typically imiquimod 3–5% which works by stimulating a local immune response [21,30]. When topical immunomodulation is unsuitable or fails, cytotoxic agents such as podophyllotoxin or trichloroacetic acid are commonly used as alternatives [23,31]. For large, widespread, or treatment-resistant warts, procedural options are cryotherapy, surgical excision, or CO_2_ laser ablation [6,32,33]. Pregnancy deserves special consideration: the physiological immunomodulation of pregnancy can favor lesion persistence, so treatment is usually deferred until after the second trimester and is most often surgical to lower risks such as premature rupture of membranes (PROM) and neonatal transmission [34].

However, it is important to remark that condyloma acuminata infection in pregnancy is not an indication for cesarean section [35].

Genital herpes caused by HSV is managed primarily with antivirals. Acyclovir, famciclovir, or valacyclovir started within 72 h of symptom onset shortens the episode and lessens severity. Therapy can be given episodically for individual outbreaks or as suppressive therapy when recurrences are frequent. Management also includes counseling and, when appropriate, testing for HIV and other sexually transmitted infections as part of comprehensive care [12].

Molluscum contagiosum is frequently managed conservatively because lesions often resolve on their own. When intervention is chosen for symptomatic, cosmetically concerning, or spreading lesions, effective options include mechanical removal by curettage, cryotherapy, or topical chemical treatment such as trichloroacetic acid [11,15].

Lipschütz ulcers are essentially treated supportively: analgesics, soothing topical emollients or creams, and careful local hygiene are the mainstays. Most cases run a self-limited course and heal without aggressive pharmacologic therapy, typically within two to three weeks [4].

#### Comparison of International Guidelines

Following the key recommendations summarized in Table 3, UK, European, and Australian guidelines converge on the routine application of high-sensitivity molecular diagnostics (principally PCR/NAAT for HSV and selective HPV-DNA testing in refractory or high-risk cases) and unanimous support for prophylactic HPV vaccination (with catch-up to mid-adult years) and, where relevant, VZV immunization. All three frameworks prioritize rapid testing to accelerate diagnosis, refine antiviral therapy, and minimize empirical antibiotic use, while aligning on first-line treatments (oral acyclovir or famciclovir for HSV; imiquimod and/or podophyllotoxin plus cryotherapy for warts).

Despite this shared foundation, marked differences remain in non-pharmacologic care pathways. Psychosocial support is formally encouraged in the UK, more selectively “advice-only” in European guidance, and fully integrated into multidisciplinary clinics in Australia. Follow-up intervals also vary from three to twelve months for HSV and tailored cytology schedules for HPV; telemedicine is explicitly endorsed in the UK and Australia, but not addressed by European panels. These divergences underscore opportunities for harmonization around patient-centered services such as routine quality-of-life screening, structured referral protocols, and flexible remote-care models alongside the established consensus on molecular testing and vaccination [36,37,38] (Table 3).

## 4. Discussion

Management of vulvar viral infections is best approached as a personalized, multidisciplinary effort that weighs not only the clinical features of each case but also the emotional and sexual impact on the patient. Prevention occupies a central role: widespread HPV vaccination has demonstrably reduced cases of condyloma acuminata and other HPV-related lesions and should be promoted as part of immunization programs. Equally important are behavioral measures, such as safe-sex education and consistent use of barrier methods that reduce transmission and recurrence [39].

Accurate and timely diagnosis guides effective care. For suspected herpes simplex virus (HSV) infection, early molecular testing (PCR/NAAT) accelerates the start of targeted antiviral therapy. In immunocompromised patients, HPV genotyping can inform dysplasia surveillance and tailor follow-up strategies.

Therapeutic options combine established antivirals and immunomodulators with symptom-directed supportive care. Molluscum contagiosum and Lipschütz ulcers, which are often self-limiting, are mainly managed with comfort measures and close observation. At the same time, new antivirals and immunotherapies are under investigation and may expand future treatment choices.

Beyond treating acute lesions, clinicians must recognize the longer-term consequences of viral infection. Persistent HSV or HPV activity and the resulting chronic inflammation have been implicated in vulvodynia: persistent viral replication and the ensuing chronic inflammatory response by promoting peripheral and central sensitization of nociceptive pathways, which can present as localized pain, allodynia, and dyspareunia [40,41,42]. For many patients, restorative care therefore includes topical anti-inflammatories and emollients to control symptoms and improve quality of life, with individualized treatment plans that consider clinical aspects.

Education and counseling are integral to any management plan. Clear information about hygiene, prevention (including consistent barrier use), and the natural history of the infections helps reduce anxiety and supports adherence to therapy. Because vulvar viral disease commonly affects sexual function, body image, and self-esteem, routine psychological support, especially for younger women, should be offered as part of comprehensive care [36,37,40,43].

Certain populations warrant heightened vigilance. Women living with HIV or those receiving immunosuppressive therapy face higher risks of recurrence and complications and therefore require more aggressive management and closer follow-up [25,26]. In all patients with recurrent infections, long-term monitoring allows clinicians to assess treatment effectiveness and intervene promptly should complications such as keratitis or precancerous changes emerge [36,37,38].

In sum, successful management of vulvar viral infections blends prevention, precise diagnosis, evidence-based therapy, symptom relief, patient education, and psychosocial support, delivered through a tailored, multidisciplinary plan that respects both the medical and emotional needs of each patient.

### 4.1. Hormonal and Noninfectious Triggers

Emerging clinical studies indicate that cyclical hormonal changes, particularly estrogen and progesterone across the menstrual cycle, during pregnancy, and around menopause, could modulate local immune defenses in the vulvar epithelium, altering susceptibility to viral reactivation. Estrogen promotes epithelial thickening and enhances the expression of tight-junction proteins, whereas progesterone has been linked to transient dampening of innate antiviral responses (e.g., reduced interferon-λ production). As estrogen levels decline in the luteal phase and again premenstrually, the mucosal barrier may become more permissive to herpes simplex virus (HSV) entry and local replication, leading to an increased risk of both initial presentation and cyclical recurrences around menses [26,44,45].

Concurrently, exposure to local irritants such as alkaline soaps, fragranced wipes, occlusive synthetic fabrics, and sexual lubricants containing irritating preservatives can disrupt the delicate vulvar microenvironment by raising pH, stripping away protective lipids, and causing low-grade inflammation. Even minor mechanical friction or microtrauma during intercourse or exercise may provide foci for viral shedding or lesion development. When these irritant exposures coincide with hormonal windows of lowered mucosal defense, patients often report clustered episodes of prodromal tingling or frank ulceration, underscoring the synergistic effect of endogenous and exogenous factors on disease activity [46].

Personalized treatment plans should therefore extend beyond standard antiviral regimens to include anticipatory guidance around predictable hormonal risk periods. For patients with catamenial (menstrual-linked) HSV outbreaks, clinicians might recommend a brief course of intensified suppressive therapy beginning several days before the expected onset of menses, coupled with barrier creams or emollients to reinforce mucosal integrity. Likewise, a thorough review of personal hygiene and sexual-wellness products can identify and eliminate potential irritants; swapping to pH-balanced, fragrance-free cleansers and choosing breathable cotton undergarments may reduce subclinical inflammation and lower recurrence rates [44,46].

Effective patient counseling should integrate education on menstrual tracking, symptom journaling, and identification of individual triggers (hormonal, chemical, or mechanical) so that women understand how to anticipate flare-ups and implement tailored prophylactic measures. By framing the management of vulvar viral infections within a biopsychosocial model that addresses endocrine fluctuations, local skin care, and lifestyle factors, clinicians empower patients to achieve better long-term control and improve quality of life [26,36,37,38,44,45].

### 4.2. Patient-Reported Outcomes and Quality of Life

Incorporating patient-reported outcomes (PROs) into both clinical practice and research protocols is vital for capturing the multidimensional burden of vulvar viral infections to include pain intensity, emotional distress, sexual dysfunction, and daily activity limitations. Validated instruments such as the Vulvar Pain Assessment Questionnaire (VPAQ) can quantify the severity, frequency, and temporal pattern of pain symptoms, while broader measures like the Dermatology Life Quality Index (DLQI) or the Female Sexual Function Index (FSFI) offer insight into how outbreaks interfere with self-image, intimacy, and psychosocial well-being. By administering these tools at baseline and at defined intervals during antiviral or adjunctive therapy, researchers can objectively track improvements in domains that matter most to patients, such as reduction in pain scores, fewer missed social or workdays, and restoration of sexual satisfaction. Moreover, embedding digital platforms or smartphone apps for real-time symptom logging enhances data granularity and helps identify individual flare-up triggers or treatment side effects. Future trials should pre-specify PRO endpoints alongside traditional virologic and dermatologic assessments, power their studies to detect clinically meaningful changes in quality-of-life scales, and report responder rates based on minimal clinically important differences. Such a patient-centered approach will ensure that novel therapies not only curb viral activity but also demonstrably improve comfort, function, and overall quality of life for those affected [47,48,49].

### 4.3. Cost-Effectiveness and Health Economics

A formal health–economic assessment comparing the per-case and population-level costs of advanced diagnostics (e.g., PCR/NAAT panels for HSV, VZV, syphilis, and other ulcer-causing pathogens) with traditional approaches (culture, serology, empirical treatment) can identify where faster, more accurate testing pays for itself by shortening time to diagnosis, reducing unnecessary treatments, and preventing complications. Likewise, cost–utility analyses of antiviral regimens, contrasting episodic versus continuous suppressive therapy, topical versus systemic formulations, and models of HPV vaccination programs (including catch-up and gender-neutral strategies) can quantify savings in downstream outcomes such as hospitalization for severe outbreaks, sequelae of chronic ulceration, and malignancies prevented by immunization. By incorporating measures like incremental cost-effectiveness ratios (ICERs), quality-adjusted life years (QALYs) gained, and budget–impact projections over short- and long-term horizons, policymakers can prioritize interventions that deliver the greatest health benefit per EUR spent. For instance, upfront investment in broad PCR/NAAT testing may be offset by reduced follow-up visits and empiric antibiotic usage, while sustained funding of HPV vaccination yields substantial reductions in anogenital wart treatment costs and cervical cancer care decades later. Ultimately, these economic evaluations will guide resource allocation toward high-value diagnostic stewardship, optimized treatment algorithms, and preventive immunization strategies, improving patient outcomes and curtailing cumulative healthcare expenditures [50].

### 4.4. Future Directions and Emerging Therapies

Novel Antiviral Agents and Immunotherapies: Advances in molecular biology are paving the way for new antiviral therapies and immunotherapies that could improve outcomes in patients with HSV and potentially other viral infections. Clinical trials investigating these agents should be closely monitored [51,52].

Advances in Molecular Diagnostics: The development of next-generation sequencing and other innovative diagnostic tools may refine the identification and typing of viral pathogens, facilitating more tailored treatment approaches.

Digital Health and Telemedicine: The use of digital health tools, including telemedicine platforms, may improve patient adherence, monitoring, and overall management of chronic viral conditions affecting the vulva.

Biomarkers of Treatment Response: Research into biomarkers that predict treatment response or disease progression will be valuable for personalizing therapy and optimizing follow-up strategies.

### 4.5. Role of Host Factors and Comorbidities

Emerging research into host determinants of vulvar viral disease promises to unlock new avenues for individualized care. Cyclical estrogen and progesterone shifts, the dramatic endocrine changes of pregnancy, and the perimenopausal transition may modulate mucosal immunity and barrier integrity in ways that influence both the intensity of initial infection and the propensity for reactivation. Future cohort studies that correlate serial measurements of sex-steroid levels with viral shedding rates or lesion severity scores could clarify exactly when and why hormonal “windows of vulnerability” occur and inform recommendations for time-tailored suppressive regimens or adjunctive topical therapies.

At the genomic level, polymorphisms in innate immune receptors (e.g., TLR2, TLR9), antiviral signaling pathways (e.g., IFNL3/4 loci), and killer-cell immunoglobulin-like receptors (KIRs) are known to affect host resistance to herpesviruses in other anatomical sites. Well-powered genetic association studies could identify patient subgroups who mount suboptimal interferon responses or who are prone to exaggerated inflammatory damage. Such findings would pave the way for precision immunomodulation, whether through targeted cytokine therapies, therapeutic vaccines, or optimized dosing of nucleoside analogues.

Immune status is another critical axis: patients with HIV, autoimmune diseases, or iatrogenic immunosuppression (e.g., post-transplant, biologic agents) exhibit both higher viral loads and more protracted healing. Longitudinal immunophenotyping tracking CD4^+^ T-cell subsets, NK-cell activity, and mucosal homing markers could reveal specific deficits that correlate with recurrence risk or poor response to standard antivirals. Interventional trials might then test whether immunorestorative strategies (e.g., low-dose IL-2, therapeutic immunoglobulins) can safely augment antiviral control in these vulnerable populations.

Finally, integrating these host-factor insights into adaptive clinical trial designs will allow real-time stratification of participants by hormonal phase, genotype, or immune profile. By powering studies to detect differential responses across these strata, investigators can refine treatment algorithms, prescribing episodic versus continuous suppressive therapy, or adding prophylactic immunomodulators, based on each patient’s unique biological signature. In this way, future work on host influences will move vulvar viral infection management decisively toward a truly personalized paradigm.

### 4.6. Interdisciplinary and Multidisciplinary Approaches

Emphasizing collaborative care among dermatologists, gynecologists, primary care physicians, and mental health specialists can improve treatment outcomes. Future guidelines should address best practices for multidisciplinary management, including patient education and counseling tailored to the psychosocial aspects of these conditions.

### 4.7. Limitations and Research Gaps

A critical appraisal of the existing literature is necessary to identify limitations and areas requiring further research. Gaps in high-quality evidence for certain diagnostic and therapeutic modalities should be acknowledged, and recommendations for future large-scale, randomized controlled trials should be prioritized.

## 5. Conclusions

Vulvar viral infections present complex clinical and psychosocial challenges that demand a patient-centered, multidisciplinary response. Prevention through HPV vaccination and safe-sex education remains the foundation for reducing disease burden, while early molecular diagnostics (PCR/NAAT) and targeted genotyping in high-risk or immunocompromised patients enable prompt, precise treatment.

First-line antiviral and immunomodulatory regimens like oral acyclovir/famciclovir for HSV and topical imiquimod or podophyllotoxin (±cryotherapy) for HPV, are complemented by supportive care for self-limiting conditions (molluscum, Lipschütz ulcers) and emerging therapies under clinical evaluation. Recognizing the role of hormonal fluctuations, local irritants, and host immune status in disease onset and recurrence allows clinicians to tailor suppressive strategies, non-pharmacologic measures, and timing of therapy for each patient.

Comprehensive management extends beyond viral suppression to address pain, sexual function, and emotional well-being. Incorporation of validated patient-reported outcome measures (e.g., VPAQ, DLQI, FSFI) and structured long-term follow-up protocols ensures that therapeutic success is measured in quality-of-life gains as well as virologic control, particularly among vulnerable groups such as immunocompromised individuals and children.

Health-economic analyses reinforce the value of upfront investment in rapid diagnostics, sustained antiviral suppression, and broad vaccination programs by demonstrating downstream savings in complication management and cancer prevention. Future research should prioritize randomized trials of novel antivirals and immunotherapies, advanced diagnostics (e.g., next-generation sequencing), digital health interventions, and biomarker-driven treatment algorithms.

Finally, harmonization of international guidelines, particularly around psychosocial support, complementary therapies, telemedicine, and multidisciplinary care pathways, will be essential to standardize best practices and optimize outcomes globally. By integrating prevention, precision diagnostics, personalized therapy, psychosocial care, and rigorous economic evaluation, clinicians and policymakers can deliver truly holistic, high-value care for individuals affected by vulvar viral infections.

## Figures and Tables

**Table 1:** Differential clinical characteristics of vulvar viral infections.

Pathology	Lesions	Symptoms	Diagnosis	First-Line Therapy
Condylomata Acuminata (HPV)	Verrucous papules, cauliflower-like plaques	Pruritus, discomfort	Clinical; colposcopy; biopsy/HPV genotyping	Imiquimod; cryotherapy; CO_2_ laser
Genital Herpes	Vesicles → painful ulcers	Pain, dysuria, fever, lymphadenopathy	PCR/NAAT; serology	Acyclovir; valacyclovir; famciclovir
Molluscum Contagiosum	Dome-shaped papules with central umbilication	Often asymptomatic	Clinical; dermatoscopy; biopsy if needed	Curettage; cryotherapy; expectant management
Lipschütz Ulcers	Deep, symmetric ulcers	Severe pain, fever, malaise	Diagnosis of exclusion	Supportive care (analgesics, hygiene, soothing agents)

Clinical Characteristics of Vulvar Viral Infections. Background color has been added to improve readability.

**Table 2:** Indications for molecular testing in vulvar viral infections.

Pathology	Molecular Test	Indication	Benefit
Genital Herpes	PCR/NAAT for HSV on lesion	Acute ulcerative lesions	Rapid and sensitive detection
Condylomata Acuminata (HPV)	HPV-DNA test	Atypical or refractory lesions; immunodeficient patients	Genotypic identification
Molluscum Contagiosum	(Not routinely used) *	Atypical cases; in immunocompromised patients	Confirmation of etiology if needed
Lipschütz Ulcers	PCR for HSV, VZV, EBV, CMV	Acute febrile ulcerative conditions	Exclusion of known viral etiologies

* Note: For molluscum contagiosum, molecular tests are not routinely used unless the clinical picture is unclear. Background color has been added to improve readability.

**Table 3:** Key Recommendations from UK, European, and Australian guidelines.

Topic	UK (BASHH 2023)	European (EADV 2024)	Australian (ASHM 2023)
Molecular Diagnosis	PCR/NAAT for HSV; HPV-DNA only if refractory	PCR/NAAT for HSV; routine HPV genotyping in immunocompromised	PCR/NAAT for HSV; HPV-DNA in atypical/recurrent warts
HPV Vaccination	Recommended up to age 25; catch-up to 45	Strongly recommended 9–26; extended to 45 in some countries	Routine 12–13 years; catch-up to 26 years
Screening and Follow-Up	Cytology every 3 years post-treatment	Cytology/VIA based on age and risk	Pap smear 12 months after treatment; then per national program
Psychosocial Support	Formal counseling referral encouraged	Advice only; referral if requested	Integrated sexual health counseling recommended
Treatment First-Line	Acyclovir/Famciclovir; Imiquimod for warts	Same; prefers podophyllotoxin over imiquimod	Same; imiquimod and cryotherapy equally recommended
Follow-Up Interval	HSV: 6 months; HPV: annually	HSV: 3–6 months; HPV: per cytology results	HSV: 3 months; HPV: per immunosuppression status
Telemedicine	Supported for follow-up of stable cases	Not specifically mentioned	Encouraged for remote consultations

## Data Availability

Data are available under reasonable request to the corresponding author.
